# Retention in Care After Transition to Adult Care for Adolescents and Young Adults With HIV: A Systematic Review and Meta-Analysis

**DOI:** 10.3389/ijph.2025.1607733

**Published:** 2025-03-24

**Authors:** Mulugeta Shegaze Shimbre, Abebe Gedefaw Belete, Tamirat Gezahegn Guyo, Wei Ma

**Affiliations:** ^1^ School of Public Health, Cheeloo College of Medicine, Shandong University, Jinan, Shandong, China; ^2^ School of Public Health, College of Medicine and Health Sciences, Arba Minch University, Arba Minch, Ethiopia; ^3^ Department of Public Health, Arba Minch College of Health Sciences, Arba Minch, Ethiopia

**Keywords:** retention in care, transition to adult care, adolescents, young adults, HIV

## Abstract

**Objective:**

To evaluate the retention rates of adolescents and young adults (aged 10–25 years) living with HIV during the transition to adult HIV care.

**Methods:**

The study involved 15 cohort studies conducted since 2015, focusing on adolescents and young adults aged 10–25 years living with HIV who transitioned to adult care. The primary outcome measured was the retention rate in care after transition. Data screening and extraction were performed using Covidence software, and the quality of included studies was assessed using the Joanna Briggs Institute tool.

**Results:**

The pooled 1-year retention rate was 81% (95% CI: 78%, 91%), while the 2-year retention rate was 69% (95% CI: 53%, 83%). Significant heterogeneity was observed between studies (I^2^ = 96.73%). Subgroup analyses revealed geographical differences, with Asia exhibiting the highest retention rates. Retrospective study designs yielded better retention outcomes.

**Conclusion:**

The findings underscore the challenges and variability in retention rates for adolescents transitioning to adult HIV care. There is a critical need for targeted interventions and improved follow-up strategies to enhance retention and meet global HIV care targets.

## Introduction

In recent years, substantial progress has been made in HIV/AIDS management, notably in increasing access to effective antiretroviral therapy (ART) and improving clinical outcomes [1, 2]. However, a persistent challenge is the effective transition of adolescents and young adults from pediatric or adolescent HIV care to adult HIV care settings [2, 3]. This transition represents a crucial stage in the healthcare trajectory of people living with HIV (PLWH), characterized by numerous complexities and potential disruptions in continuity of care [4–6].

Adolescents and young adults living with HIV face unique challenges during this transition period, such as navigating changes in healthcare providers, environments, and support systems, in addition to managing evolving psychosocial and developmental needs [7]. The successful retention of this vulnerable population in adult HIV care is critical to ensuring optimal health outcomes, preventing treatment interruptions, and reducing the risk of virologic failure, and ongoing HIV transmission [8, 9]. However, retention in care during this transition is a significant challenge, particularly in sub-Saharan countries [2].

Evidence on the transition of adolescents and young adults with HIV to adult care shows regional variations in retention rates, ranging from 37% to 94.7% [10]. Retention in adult care is generally lower than in pediatric care. In high-income countries, more than 75% of patients are retained in care approximately 4 years post-transition [2, 11], with rates of 89%–94% in the United States [4] [4, 12, 13], 83% in Poland [14], and 86% in the Netherlands [15]. South African studies reported retention rates of 80%–92% among adolescents transitioning to adult care [16–18], while a single-center study in the Sahel Region of West Africa, reported a retention rate of 95.8% [19]. In Uganda, retention decreased from 90% to 84% within 1–3 years of transition [20]. In contrast, in low- and middle-income countries, retention drops to 55% after adolescents and young adults transition to adult care [10].

After the transition, reduced retention in care has a disproportionate impact on the health of adolescents and young adults [2]. A study from Canada found that health outcomes for adolescents transitioning to adult care dropped to 75% [21]. A study from the United States reported that after transition to adult care, retention rates decreased from 89% in the first year to 56% after 2 years, impacting adolescent health outcomes such as low viral load suppression and CD4 count [4]. Zanoniet al. further supported this, showing that a significant proportion of adolescents and young adults diagnosed with HIV are not adequately retained in care, resulting in poor health outcomes [22].

Moreover, the existing literature often lacks consistency in the definition and the measurement of healthcare retention, leading to variability in reported retention rates and hindering meaningful comparisons across studies [19, 20, 23]. Retention is defined in different ways, often based on maintaining regular visits within the first 1–3 years, attending a minimum number of visits per year, or staying in care without gaps [22, 24]. This inconsistency poses a challenge in accurately assessing the extent of the retention gap and identifying effective strategies to improve healthcare continuity post-transition.

This review and meta-analysis will contribute to the existing literature by providing a comprehensive synthesis of empirical evidence on healthcare retention among adolescents and young adults transitioning to adult HIV care. By synthesizing data from various studies, this review seeks to identify retention rates and elucidate disparities across regions. The findings will inform clinical practice, policy development, and future research endeavors aimed at improving healthcare delivery and outcomes for this vulnerable population.

## Methods

### Protocol and Registration

This systematic review and meta-analysis adheres to the reporting guidelines of the Preferred Reporting Items for Systematic Reviews and Meta-Analyses (PRISMA) [25]. The protocol of the systematic review and meta-analysis was registered in the International Prospective Register of Systematic Reviews (PROSPERO) database with (https://www.crd.york.ac.uk/prospero/display_record.php? ID = CRD42024533269).

### Data Sources and Search Strategies

A comprehensive search was conducted in five selected databases, namely PubMed, EMBASE, Scopus, Web of Science, and Cochrane Central Library. In addition, a systematic search was conducted in Google, Google Scholar, and institutional repositories to access gray literature. A combination of keywords and controlled vocabularies such as Medical Subject Headings (MeSH) in PubMed and Emtree in Embase was used in the searching process. The terms (Retention, retain*, “Retention in Care,” Adolescent*, Young adult*, Youth*, “Living with HIV,” “HIV positive,” “HIV infected,” Transition, Transfer, Link, Movement, “Transition to Adult Care,” “Patient Transfer”) with Boolean operators (OR, AND, and NOT), field codes, wildcards, brackets, and quotation marks were used to search in each database (Supplementary Table S1).

### Inclusion and Exclusion Criteria

This systematic review and meta-analysis included studies that met specific inclusion criteria. The study population consisted of adolescents and young adults aged 10–25 years living with HIV who had transitioned from pediatric to adult HIV care. Additionally, only studies with cross-sectional and cohort study designs were considered. Finally, only cohort studies that fulfilled the eligibility criteria were included. The review focused on studies conducted after January 2015 and required that all included studies be published in the English language.

Conversely, studies were excluded if they met any of the following conditions: first, adolescents and young adults aged 10–25 years living with HIV who had not transitioned from pediatric to adult HIV care were excluded, particularly those with missing information about their transition. Second, studies that utilized designs other than cross-sectional and cohort studies were not included. Third, all studies conducted before January 2015 were excluded. Fourth, studies published in languages other than English were not considered. Finally, studies that were not available in full-text or had insufficient data for analysis were also excluded. The reasons for excluding primary studies from the review and analysis are presented in Supplementary Table S2.

### Study Selection

All articles retrieved from the databases and other sources were downloaded and exported into the Covidence software [26]. Covidence is well known for its user interface and functionalities that promote collaboration among reviewers. It facilitates the removal of records of aids, in screening titles abstracts, and full texts efficiently. Additionally, we used covidence for data extraction, quality of articles evaluation, and generation of PRISMA flow diagrams.

For this research project, two independent reviewers (MS and AG) screened titles, abstracts, and full texts. The screening process was streamlined using Covidence’s features such as blinded assessments and conflict resolution mechanisms. Any disagreements between the reviewers were resolved by a reviewer (TG) who made decisions to ensure consistency and accuracy, in the review process.

### Data Extraction and Study Quality Assessment

Data were extracted from articles that met the inclusion criteria. We used Covidence to extract data containing the following categories: Name of the Author, Year of publication, Study Setting, Study Design, Study Population, Total number of transitions, Total number of Retention, and Retention rate. The data were extracted by two reviewers together to facilitate data extraction and minimize errors. We used The Joanna Briggs Institute (JBI) critical appraisal tool to assess the quality of our papers [27, 28]. The tool has eleven items to measure the quality of cohort studies. An article with a total JBI quality score greater than 50% was considered good quality and was included in our data analysis (Supplementary Table S3).

### Data Synthesis and Analysis

The extracted data were exported to Stata version 18 for data synthesis and statistical analysis. The characteristics of the extracted data were presented in the table and a summary of the pooled estimate was presented graphically in a forest plot. The random effects model was used because of the high level of heterogeneity between studies. The presence of statistical heterogeneity among the included studies was determined using the Higgins I2 statistics and the Cochran-Q test, with I2 values of 25%, 50%, and 75% being low, medium, and high respectively. Additionally, subgroup analyses by geographic region, study design, publication, median age at the time of care transition, and population type were performed to examine the variation in estimates among the categories and sensitivity analyses were also conducted to check the effect of a single study on the overall pooled estimate. The Freeman-Tukey double arcsine transformation was used to generate the retention rate with a 95% confidence interval and to stabilize the variance of each study’s proportion [29]. Publication bias was assessed graphically using a funnel plot and statistically using Egger’s regression test. The results were presented using text, figures, and tables.

## Results

### Search Results

**FIGURE 1:**
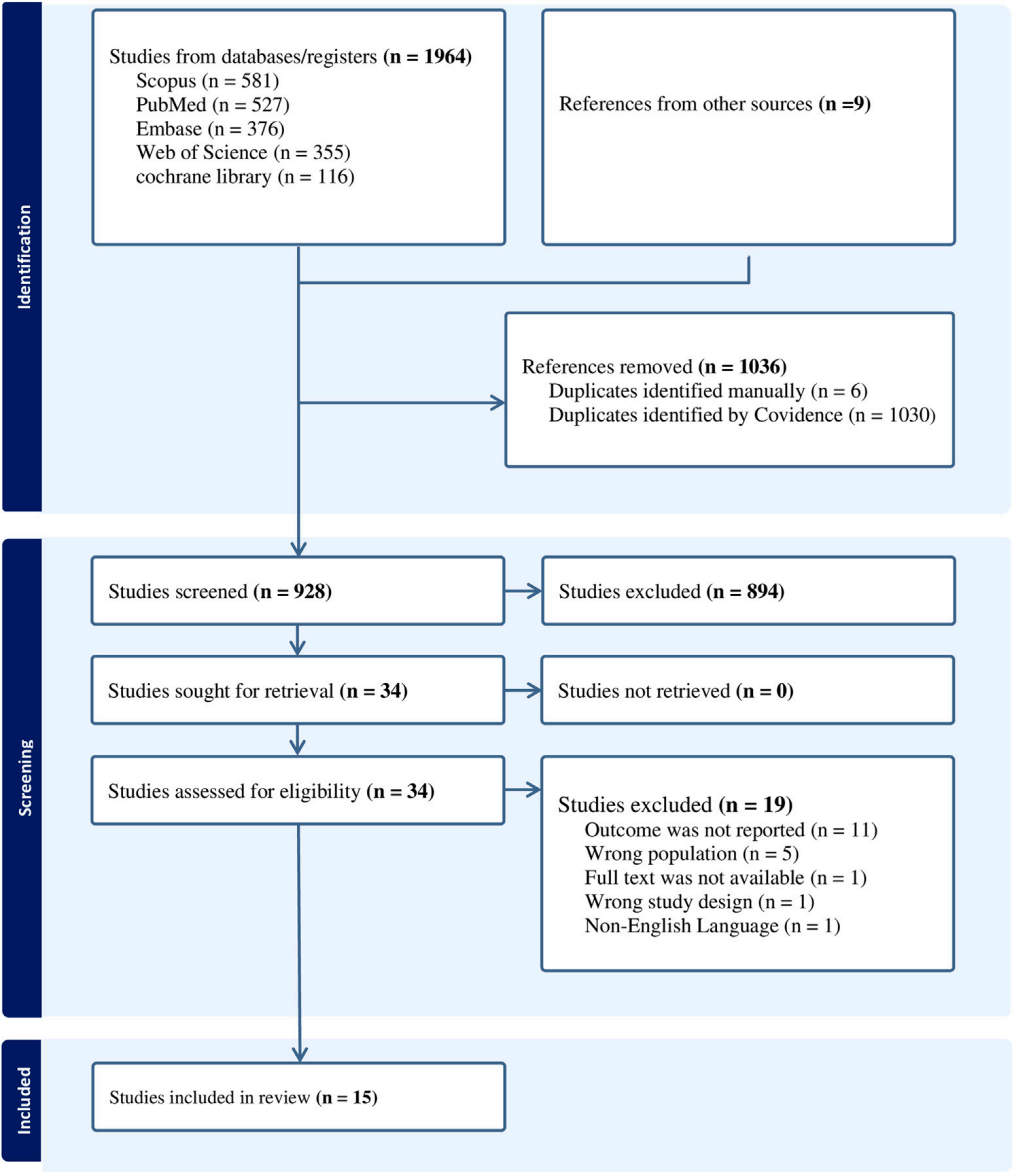
Flow chart of preferred reporting items for systematic review and meta-analysis (PRISMA): a systematic review and meta-analysis (worldwide, 2024).

A total of 1964 studies (1955 studies from five databases and 9 studies from other sources) were identified. Of the total studies 1,036 studies were identified as duplicates (1,030 studies by COVIDENCE software (https://www.covidence.org/) and 6 studies manually) and 928 studies passed for title and abstract screening. Of the studies that passed the title and abstract screening 894 studies were excluded as irrelevant and 34 studies passed the full-text screening. Finally, out of the 34 studies, 15 studies were included in the systematic review and meta-analysis, with the remaining 19 studies excluded (eleven of them did not publish the results, five of them had the wrong population, one study did not have the full text, one had the wrong study design, and one was not in English) (Figure 1).

### Study Characteristics

A total of fifteen studies that met the eligibility criteria were used for this systematic review and meta-analysis with a total of 13,764 study participants. Five studies were conducted in the United States [Griffith, 2019 #11; Hussen, 2017 #12; Hussen, 2023 #19; Nassau, 2022 #17; Tanner, 2018 #10], seven studies in Africa [Abebe, 2021 #8; Castelnuovo, 2018 #20; Davies, 2017 #21; Meloni, 2020 #9; Ouedraogo, 2024 #18; Tsondai, 2020 #16; Zanoni, 2021 #14], one study in Canada [Kakkar, 2016 #15], one study in Asia [Rungmaitree, 2022 #7], and one study in the United Kingdom [Foster, 2020 #13]. Of these four were prospective cohort studies and eleven were retrospective cohort studies. Nine studies were published in or after 2020 and the remaining six were published before 2020. The studies were conducted among adolescents and young adults aged 10–25 years using sample sizes ranging from 19 to 11,283 subjects. Twelve studies were conducted among adolescents and three of them among young adults. Thirteen studies reported the median age at transition of care, ranging from 12.9 years (11.4–15.3) to 24.4 years [24, 25]. The studies included a total of 13,756 individuals with 6,373 (46.3%) male subjects and 7,383 (53.7%) female subjects (Table 1).

**TABLE 1:** Summary of the characteristics of studies included in the systematic review and meta-analysis: a systematic review and meta-analysis (worldwide, 2024).

S.No	Author and year of publication	Study setting	Study design	Sample size	Population	One year retention	Two-year retention	Transition (total)	Gender, number (percent)	Age at transition
1	Supattra Rungmaitree, 2022 [7]	Thailand	Retrospective cohort	101	AYHIV 18-25	93	88	101	Male 46 (45.5%), Female 55 (54.5%)	20 (19,21)
2	Workeabeba Abebe, 2021 [8]	Ethiopia	Retrospective cohort	151	AYA 15-20	132		151	Male 87 (57.6%), Female 64 (42.4%)	18 (17,19)
3	Seema T. Meloni, 2020 [9]	Nigeria	Retrospective cohort	58	AYA 10-18	50		58	Male 28 (49.2%), Female 30 (50.8%)	17 (16, 17.9)
4	Amanda E. Tanner, 2018 [24]	USA	Prospective cohort	132	AYA 21-24	49		132	Male 100 (75.7%), Female 32 (24.3%)	22.5 (21, 24)
5	David Griffith, 2019 [12]	USA	Retrospective cohort	89	AYA 18-25	79		89	Male 34 (38%) Female 55 (62%)	
6	Sophia A. Hussen, 2017 [4]	USA	Retrospective cohort	70	AYA 21-25	62	38	70	Male 45 (62.5%), Female 25 (37.5%)	23.8 (22,24.8)
7	Caroline Foster, 2020 [30]	UK	Retrospective cohort	180	AYA 15-25	158		180	Male 92 (44.3%), Female 88 (55.7)	17.5 (15.2, 20.4)
8	Brian C. Zanoni, 2021 [22]	S/Africa	Prospective cohort	19	AYA 15-19	11		19	Male 11 (58%), Female 8 (42%)	16.5 (15.8, 16.9)
9	Fatima Kakka, 2016 [21]	Canada	Retrospective cohort	25	AYA 18-25	19		25	Male 10 (40%), Female 15 (60%)	
10	Priscilla R Tsondai, 2020 [31]	Six S/African countries (Lesotho, Malawi, Mozambique, South Africa, Zambia and<!--Soft-enter Run-on-- > Zimbabwe	Prospective cohort	11,283	AYA 16-22	9,473		11,283	Male 5,354 (47.5%) female 5,929 (52.5%)	19 (16, 22)
11	Tanner Nassau, 2022 [32]	USA	Retrospective cohort	232	AYA 18-25	137	105	232	Male 175 (75.4%), Female 57 (24.6%)	21.5 (18, 25)
12	Paul Ouedraogo, 2024 [19]	Burkina Faso	Retrospective cohort	73	AYA 13-25	70	68	73	Males 39 (53.4%), Female 34 (46.6%)	17 [16, 18]
13	Sophia A.Hussen, 2023 [23]	USA	Prospective cohort	70	AYA 20-25	51	45	62	Male 55 (88.6%), Female 7 (11.3%)	24.4 [24, 25]
14	Barbara Castelnuovo, 2018 [20]	Uganda	Retrospective cohort	907	AYA 18-23	Data not available	429	907	Male 123 (13.5%), Female 784 (86.5%)	21 (20, 22)
15	Mary-Ann Davies, 2017 [17]	South Africa	Retrospective cohort	374	AYA 10-19	310	274	374	Male 174 (46.5%), Female 200 (53.5%)	12.9 (11.4–15.3)

### Pooled One-Year Retention Rate After Transition to Adult HIV Care

**FIGURE 2:**
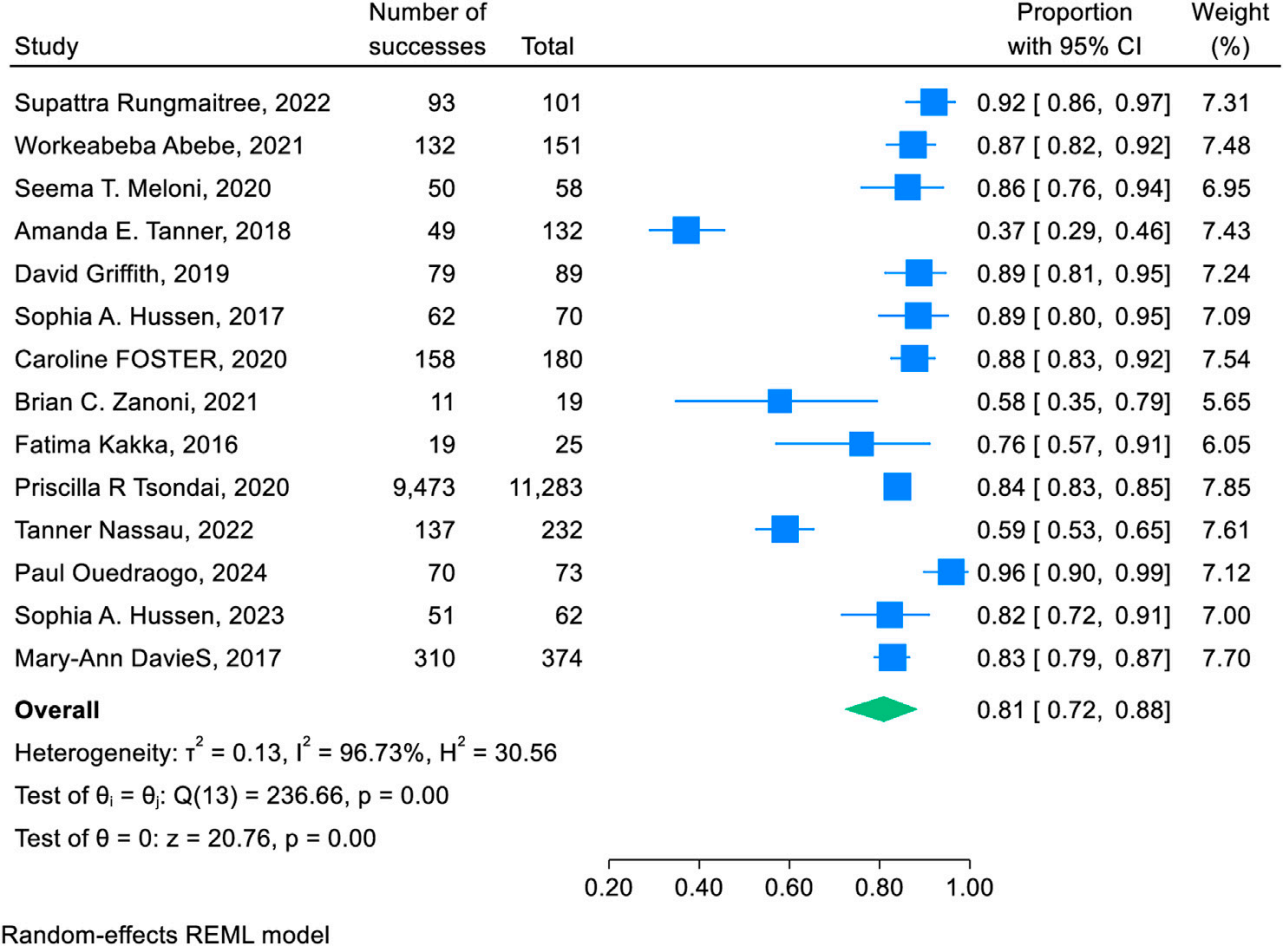
Forest plot of the retention rate of 1 year after transition to adult HIV care: a systematic review and meta-analysis (worldwide, 2024).

Fourteen studies were used to estimate a pooled 1-year retention rate of adolescents and young adults after the transition to adult HIV care. The pooled retention rate was 81% (95% CI: 72%, 88%) with point retention rates ranging from 37% (95% CI: 29%, 46%) to 92% (95% CI: 86%, 97%). There was a statistically significant higher degree of heterogeneity among the studies (I2 = 96.73%, P < 0.001). Therefore we used a random effects model to adjust for the heterogeneity between studies (Figure 2).

### Pooled Two-Year Retention Rate After Transition to Adult HIV Care

**FIGURE 3:**
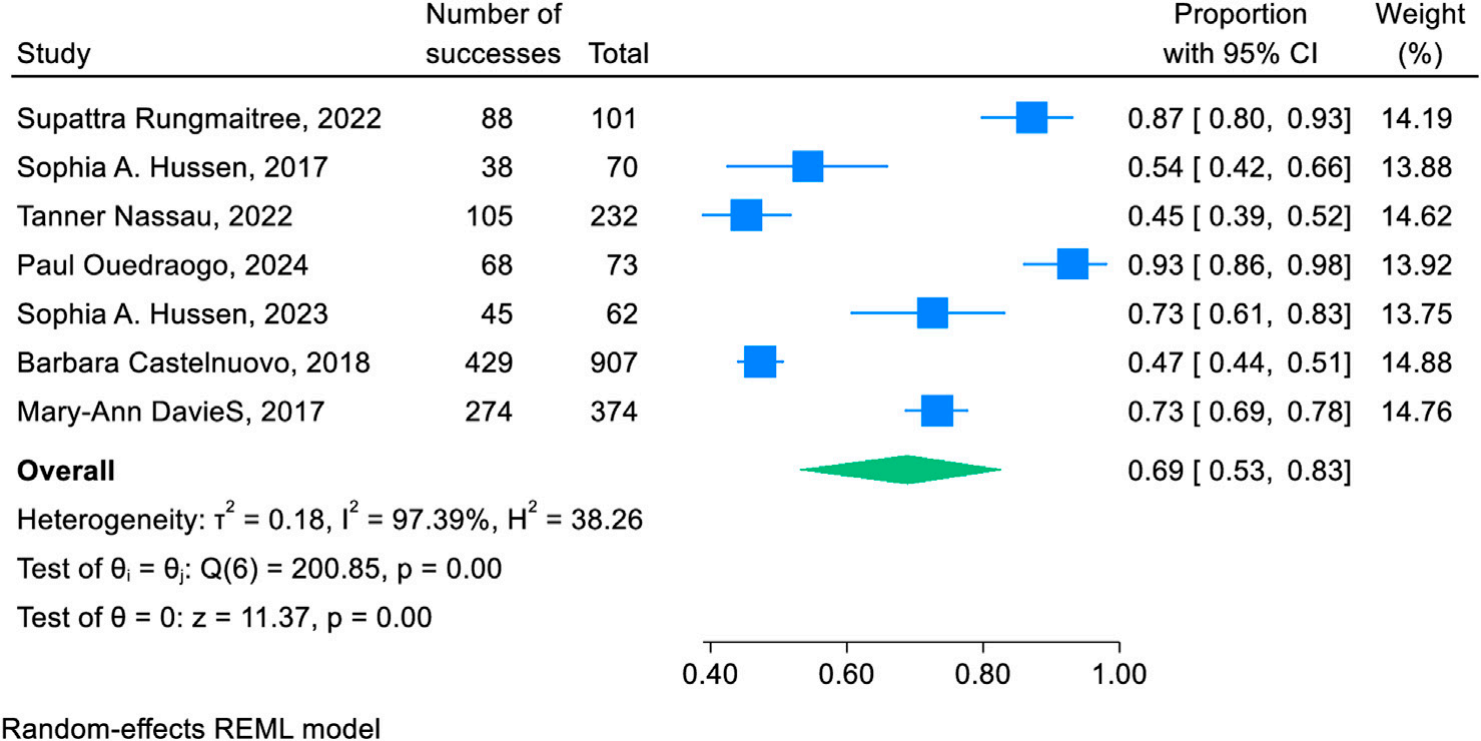
Forest plot of the retention rate of 2 years after transition to adult HIV care: a systematic review and meta-analysis (worldwide, 2024).

Seven studies were used to estimate the pooled 2-year retention rate of adolescents and young adults after transition to adult HIV care. The pooled retention rate was 69% (95% CI: 53%, 83%) with point retention rates ranging from 45% (95% CI: 39%, 52%) to 93% (95% CI: 86%, 98%). There was a statistically significant higher degree of heterogeneity among the studies (I2 = 97.39%, P < 0.001). Therefore we used a random effects model to adjust for the heterogeneity between studies (Figure 3).

### Sub-Group Analysis and Meta-Regression

Sub-group analysis was performed by geographic regions (Africa, Asia, Europe, and North America), study design (Prospective or Retrospective), year of publication (before 2020 and after 2020), median age of transition (before 20 years or after 20 years) and population type (adolescents or young adults) (Supplementary Figures S1–S10). A statistically significant difference was observed only by geographical region for both the 1-year and 2-year retention rates (P < 0.08 and P < 0.01, respectively). The pooled 1-year retention rate was 85% (95% CI: 78%, 91%) in Africa, 87% (95% CI: 80%, 93%) in Asia, 88% (95% CI: 83%, 92%) in Europe, and 73% (95%CI: 55%, 88%) in North America while the pooled 2-year retention rate was 73% (95%CI: 44%, 94%) in Africa, 92% (95%CI: 86%, 97%) in Asia, and 57% (95%CI: 41%, 72%) in North America (Supplementary Figures S1, S2).

Furthermore, meta-regression was performed on both 1-year and 2-year retention rates to determine the effect of covariates on the pooled estimates and only study design was found to be a significant factor affecting the pooled estimates for 1-year retention rates. Retrospective studies had a positive relation with the pooled estimates (with coefficient = 0.429, and P-value = 0.035) (Supplementary Table S4).

### Publication Bias and Sensitivity Analysis

**FIGURE 4:**
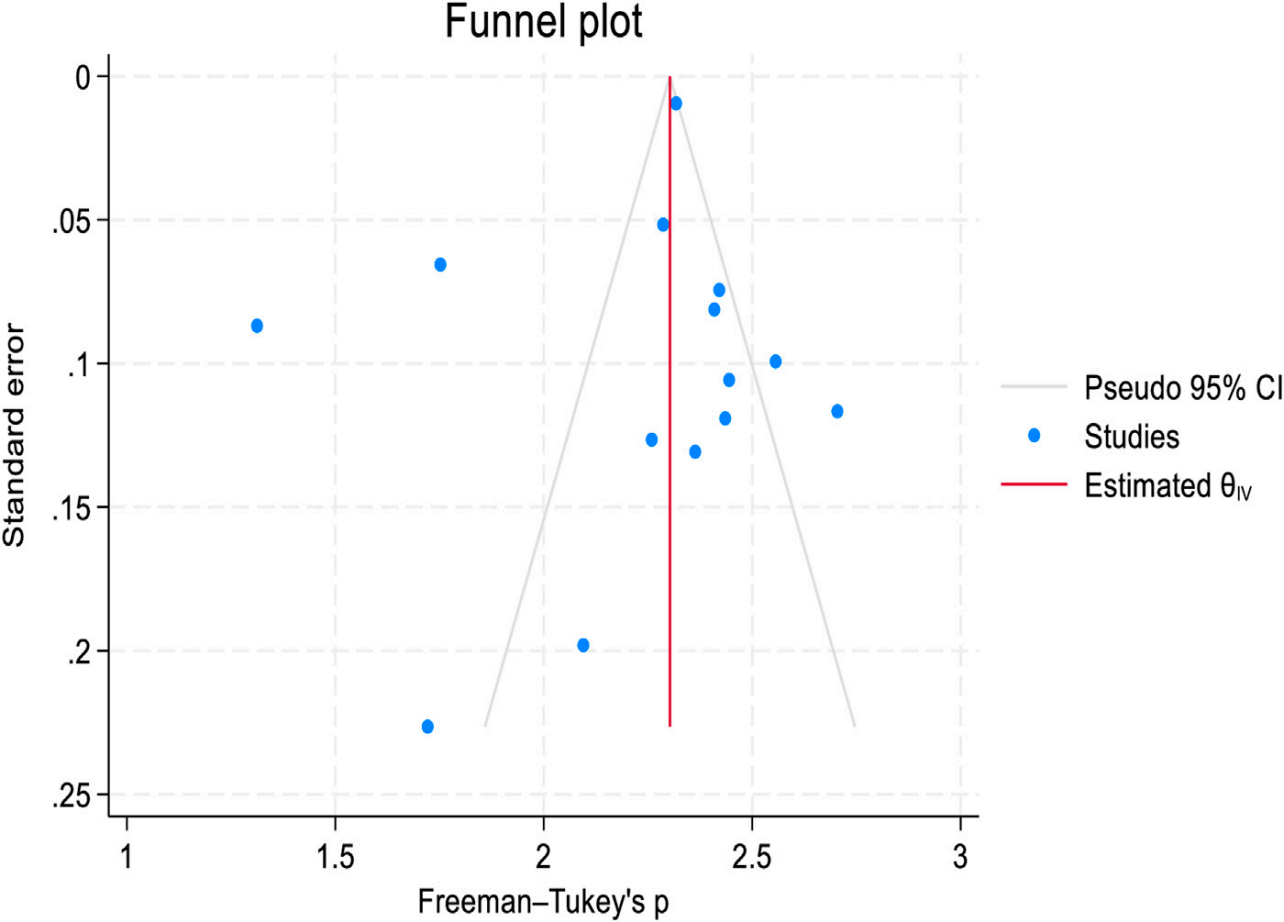
Funnel plot to assess publication bias in the 1-year retention rate after transition to adult HIV care: a systematic review and meta-analysis (worldwide, 2024).

**FIGURE 5:**
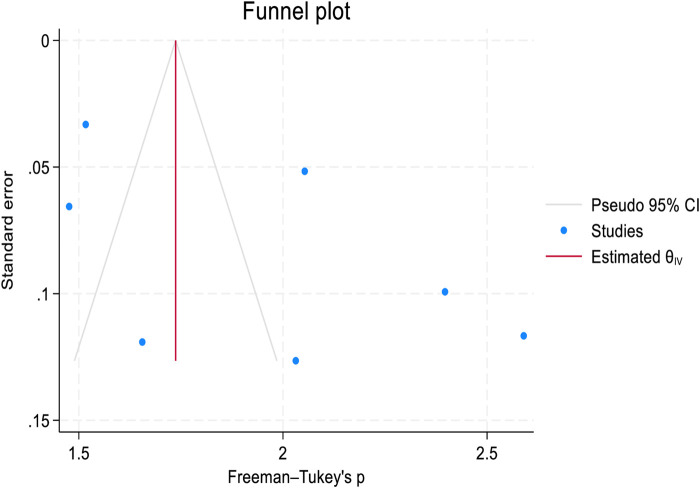
Funnel plot to assess publication bias in the 2-year retention rate after transition to adult HIV care: a systematic review and meta-analysis (worldwide, 2024).

Publication bias was assessed graphically using a funnel plot and statistically using Egger’s test for both 1-year and 2-year retention rates. Although a mild asymmetry was observed in the funnel plot (Figures 4, 5). Egger’s test showed the absence of publication bias with a p-value of (P = 0.6393 and P = 0.1878, in 1-year and 2-year retention rates respectively (Supplementary Tables S5–S6). Sensitivity analysis was performed to assess the effect of a single study on the pooled analysis. The results indicate that no single study significantly impacts the pooled estimates for both 1-year and 2-year retention rates, meaning that the overall pooled estimates remain stable across studies (Supplementary Figures S11, S12).

## Discussion

The primary objective of our study was to examine the 1-year and 2-year retention rates immediately following the transition to adult HIV care in order to assess the effect of transition on retention. In this study, fifteen studies met the eligibility criteria and were included in the systematic review and meta-analysis to determine the global pooled retention rate of adolescents and young adults transitioning to adult HIV care. Fourteen studies were used to estimate the pooled 1-year retention rate, and seven studies were used to estimate the pooled 2-year retention rate. The pooled 1-year retention rate was 81%, and the pooled 2-year retention rate was 69% after transition to adult HIV care. However, many of the studies cited earlier that focused on retention rates, did not specify whether they were conducted immediately after transition. Additionally, some of these studies were conducted before 2015, while others combined adult and adolescent populations, leading to their exclusion from our analysis. We also excluded the systematic review from our discussion, as it primarily focused on adult populations, which were not relevant to the scope of our study.

The current study reported that the pooled prevalence of the 1-year retention rate among adolescents and young adults transitioning to adult HIV care was over 80%. This rate is higher than findings from studies conducted in South Africa (58%) [22], and the United States (59%) [32]. It also slightly exceeds findings from retrospective cohort studies conducted in Canada (76%) [21], and South Africa, where a 74% retention rate was observed 12 months after transition to adult care [31].

In contrast, the pooled 1-year retention rate from this study is lower than rates reported in retrospective cohort studies from Ethiopia (87.4%) [8], Nigeria (87.7%) [9], Burkina Faso (95.8%) [19], Thailand (92.1%) [7], and the United States (89%) [4, 12]. This retention rate of over 80% may be due to initial support structures such as orientation sessions, transition counseling, and close follow-up by healthcare providers. These forms of support may temporarily improve retention as adolescents and young adults transition to adult care.

The pooled 2-year retention rate identified in this review was 69% following transition to adult HIV care. This rate is higher than findings from retrospective cohort studies conducted in the United States (56%) [4] and lower than results from studies conducted in Kampala, Uganda (85%) [20], South Africa (84%) [17], and Thailand (92.1%) [7]. This variation may be due to differences in how retention is defined and measured, along with disparities in healthcare system practices [22, 24]. Additionally, differences may stem from variations in HIV prevalence, healthcare infrastructure, and levels of HIV-related stigma across countries, all of which may influence post-transition retention rates [4, 8, 22, 24, 31]. The pooled 2-year retention rate of 69% underscores the challenges of maintaining long-term engagement in adult care. While initial support mechanisms may aid retention during the first year after the transition, maintaining consistent engagement over time proves increasingly difficult, particularly in regions with significant structural and societal barriers.

Our study found statistically significant variation in retention rates at one and 2 years after transition to adult HIV care across different geographic regions. Asia and Europe had the highest 1-year retention rates, while Asia had the highest 2-year retention rates. These differences may be attributed in part to the geographic distribution of the studies included in the review, with seven studies conducted in Africa, six in North America, one in Europe, and one in Asia. The limited representation of Asia, with only one study, highlights a significant gap in the data, leaving large, high-risk populations in regions such as the Middle East, Bangladesh, Malaysia, Indonesia, and Pakistan inadequately studied.

In the current study, only study design was found to be significantly associated with both the 1-year and 2-year retention rates. The retention rate was higher in studies that used retrospective study designs to determine 1-year and 2-year retention rates among adolescents and young adults after the transition to adult HIV care globally. This might be due to the fact that retrospective studies used long follow-up periods and nearly three-quarters of the studies included in the review were retrospective in design. Moreover, other study designs such as the prospective cohort study were poorly represented in the review.

### Limitations of the Study

This systematic review and meta-analysis has several limitations. The original studies that were included had varying follow-up durations, leading to differences in outcomes related to healthcare retention. Additionally, differences in HIV care guidelines and recommendations between studies may have influenced treatment outcomes and retention rates. Furthermore, this review was potentially affected by bias due to the dominance of retrospective study designs, with limited representation from other designs, such as prospective cohort studies.

### Conclusion

More than four out of five adolescents and young adults were retained in HIV care 1 year after transition to adult care, with retention dropping to two-thirds by the second year. This highlights the need for further efforts to achieve the ambitious 95-95–95 targets of the global ART program. Given changes in treatment eligibility criteria over time and varying definitions of healthcare retention, it is critical to standardize how retention is operationalized. Additionally, close and frequent follow-up, along with robust mechanisms to trace defaults, is strongly recommended to improve retention rates.

## Data Availability

The datasets used during the current systematic review and meta-analysis are available from the corresponding author upon reasonable request.
