# Supplementary material for: Retention in Care After Transition to Adult Care for Adolescents and Young Adults With HIV: A Systematic Review and Meta-Analysis
Source: Int J Public Health. 2025 Mar 24;70:1607733. doi: 10.3389/ijph.2025.1607733 (PMC11972947; doi:10.3389/ijph.2025.1607733)
Supplement: Supplementary file 1 [file Table1.docx]

**Supplementary Table 1:** Full Search strategies used in the systematic review and meta-analysis (worldwide, 2024).

| No | Name of database | Search strings |
| --- | --- | --- |
|  | PubMed | (("Retention in Care"[MeSH Terms] OR "Continuity of Patient Care"[MeSH Terms] OR ("Retention"[Title/Abstract] OR "retain*"[Title/Abstract] OR "retention rate*"[Title/Abstract] OR "Retention in Care"[Title/Abstract] OR "Retention in HIV care"[Title/Abstract] OR "sustain*"[Title/Abstract] OR "transition success*"[Title/Abstract] OR "continuity of care"[Title/Abstract] OR "healthcare continuity*"[Title/Abstract] OR "health care continuity*"[Title/Abstract] OR "health outcome*"[Title/Abstract] OR "healthcare outcome*"[Title/Abstract] OR "health care outcome*"[Title/Abstract] OR "transition outcome*"[Title/Abstract] OR "engagement in care"[Title/Abstract])) AND ("Adolescent"[MeSH Terms] OR "Young Adult"[MeSH Terms] OR "Adult Children"[MeSH Terms] OR ("adolescent*"[Title/Abstract] OR "young"[Title/Abstract] OR "young patient*"[Title/Abstract] OR "young female*"[Title/Abstract] OR "young girl*"[Title/Abstract] OR "young woman"[Title/Abstract] OR "young women"[Title/Abstract] OR "young man"[Title/Abstract] OR "young men"[Title/Abstract] OR "young adult*"[Title/Abstract] OR "adolescent and young adult*"[Title/Abstract] OR "adolescents and young adult*"[Title/Abstract] OR "youth*"[Title/Abstract])) AND ("HIV Seropositivity"[MeSH Terms] OR ("Living with HIV"[Title/Abstract] OR "HIV positive"[Title/Abstract] OR "HIV infected"[Title/Abstract] OR "infected with HIV"[Title/Abstract] OR "In HIV care"[Title/Abstract] OR "with HIV"[Title/Abstract])) AND ("transition to adult care"[MeSH Terms] OR "Patient Transfer"[MeSH Terms] OR ("Transition"[Title/Abstract] OR "transit*"[Title/Abstract] OR "transition to adult care"[Title/Abstract] OR "health care transition"[Title/Abstract] OR "HIV care transition"[Title/Abstract] OR "shift"[Title/Abstract] OR "Transfer"[Title/Abstract] OR "Transfer to adult care"[Title/Abstract] OR "move*"[Title/Abstract] OR "link*"[Title/Abstract] OR "link to care"[Title/Abstract] OR "HCT"[Title/Abstract]))) AND ((english[Filter]) AND (2015:2024[pdat])) |
|  | Embase | (exp retention in care/ OR (Retention OR retain* OR “Retention rate*” OR “retention in care” OR “Retention in HIV care” OR Sustain* OR “Transition success*” OR “continuity of care” OR “Healthcare continuity*” OR “Health care continuity*” OR “health outcome*” OR “Healthcare outcome*” OR “Health care outcome*” OR “transition outcome*” OR "engagement in care").ab,kw,ti.) AND (exp adolescent/ OR exp young adult/ OR exp adult child/ OR (Adolescent* OR young OR “young patient*” OR “young female*” OR “young girl*” OR “young woman” OR “young women” OR “young man” OR “ young men” OR “Young adult*” OR “Adolescent and young adult*” OR “Adolescents and young adult*” OR youth*).ab,kw,ti.) AND (exp Human immunodeficiency virus infected patient/ OR (“Living with HIV” OR “HIV positive” OR “HIV infected” OR “infected with HIV” OR “In HIV care” OR “with HIV”).ab,kw,ti.) AND (exp transition to adult care/ OR (Transition OR Transit* OR “Transition to adult care” OR “health care transition” OR “HIV care transition” OR shift OR Transfer OR “Transfer to adult care” OR Move* OR link* OR “link to care” OR “HCT”).ab,kw,ti.) |
|  | Scopus | ( TITLE-ABS-KEY ( retention OR retain* OR "Retention rate*" OR "retention in care" OR "Retention in HIV care" OR sustain* OR "Transition success*" OR "continuity of care" OR "Healthcare continuity*" OR "Health care continuity*" OR "health outcome*" OR "Healthcare outcome*" OR "Health care outcome*" OR "transition outcome*" OR "engagement in care" ) OR INDEXTERMS ( "retention in care" OR "continuity of patient care" ) ) AND ( TITLE-ABS-KEY ( adolescent* OR young OR "young patient*" OR "young female*" OR "young girl*" OR "young woman" OR "young women" OR "young man" OR " young men" OR "Young adult*" OR "Adolescent and young adult*" OR "Adolescents and young adult*" OR youth* ) OR INDEXTERMS ( "adolescent" OR "young adult" OR "adult children" OR "adult child" ) ) AND ( TITLE-ABS-KEY ( "Living with HIV" OR "HIV positive" OR "HIV infected" OR "infected with HIV" OR "In HIV care" OR "with HIV" ) OR INDEXTERMS ( "hiv seropositivity" OR "human immunodeficiency virus infected patient" ) ) AND ( TITLE-ABS-KEY ( transition OR transit* OR "Transition to adult care" OR "health care transition" OR "HIV care transition" OR shift OR transfer OR "Transfer to adult care" OR move* OR link* OR "link to care" OR "HCT" ) OR INDEXTERMS ( "transition to adult care" OR "patient transfer" ) ) AND PUBYEAR > 2014 AND PUBYEAR < 2025 AND ( LIMIT-TO ( DOCTYPE , "ar" ) ) AND ( LIMIT-TO ( LANGUAGE , "English" ) ) |
|  | Web of Science | (TS=(((Retention OR retain* OR “Retention rate*” OR “retention in care” OR “Retention in HIV care” OR Sustain* OR “Transition success*” OR “continuity of care” OR “Healthcare continuity*” OR “Health care continuity*” OR “health outcome*” OR “Healthcare outcome*” OR “Health care outcome*” OR “transition outcome*” OR "engagement in care") AND (Adolescent* OR young OR “young patient*” OR “young female*” OR “young girl*” OR “young woman” OR “young women” OR “young man” OR “ young men” OR “Young adult*” OR “Adolescent and young adult*” OR “Adolescents and young adult*” OR youth*) AND (“Living with HIV” OR “HIV positive” OR “HIV infected” OR “infected with HIV” OR “In HIV care” OR “with HIV”) AND (Transition OR Transit* OR “Transition to adult care” OR “health care transition” OR “HIV care transition” OR shift OR Transfer OR “Transfer to adult care” OR Move* OR link* OR “link to care” OR “HCT”)))) AND (DT==("ARTICLE") AND LA==("ENGLISH") AND LA==("ENGLISH") AND DT==("ARTICLE") AND PY==("2024" OR "2023" OR "2022" OR "2021" OR "2020" OR "2019" OR "2018" OR "2017" OR "2016" OR "2015")) |
|  | Cochrane | ([mh "retention in care"] OR [mh "continuity of patient care"] OR (Retention OR retain* OR Retention NEXT rate* OR “retention in care” OR “Retention in HIV care” OR Sustain* OR Transition NEXT success* OR “continuity of care” OR Healthcare NEXT continuity* OR Health NEXT care NEXT continuity* OR health NEXT outcome* OR Healthcare NEXT outcome* OR Health NEXT care NEXT outcome* OR transition NEXT outcome* OR "engagement in care"):ti,ab,kw) AND ([mh adolescent] OR [mh "young adult"] OR [mh "adult children"] OR (Adolescent* OR young OR young NEXT patient* OR young NEXT female* OR young NEXT girl* OR “young woman” OR “young women” OR “young man” OR “young men” OR Young NEXT adult* OR Adolescent NEXT young adult* OR Adolescents NEXT young adult* OR youth*):ti,ab,kw) AND ([mh "hiv seropositivity"] OR (“Living with HIV” OR “HIV positive” OR “HIV infected” OR “infected with HIV” OR “In HIV care” OR “with HIV”):ti,ab,kw) AND ([mh "transition to adult care"] OR [mh "patient transfer"] OR (Transition OR Transit* OR “Transition to adult care” OR “health care transition” OR “HIV care transition” OR shift OR Transfer OR “Transfer to adult care” OR Move* OR link* OR “link to care” OR “HCT”):ti,ab,kw) |

**Supplementary Table 2:** The reasons behind the excluded primary studies in the systematic review and meta-analysis (worldwide, 2024).

| No | Author | Title | Link | Reason |
| --- | --- | --- | --- | --- |
| 1. | Ilaria Izzo, 2018 | Perinatally HIV-Infected Youths After Transition from Pediatric to Adult Care, a Single-Center Experience from Northern Italy. | Https://dx.doi.org/10.1089/AID.2017.0120 | The outcome was not recorded |
| 2. | Angela Carvalho Freitas, 2019 | HIV-infected youths transitioning from pediatric to adult outpatient care in a teaching tertiary care hospital in São Paulo city, Brazil. | Https://dx.doi.org/10.1016/j.bjid.2019.07.004 | The outcome was not recorded |
| 3. | Ambra Righetti, 2015 | Transitioning HIV-infected children and adolescents into adult care: an Italian real-life experience. | Https://dx.doi.org/10.1016/j.jana.2015.05.003 | Wrong population |
| 4 | David Aguilera-Alonso, 2021 | Clinical, Immunological, and Virological Outcomes Among Youths With Perinatal HIV After Transition to Adult Units in Spain From 1997 to 2016. | Https://dx.doi.org/10.1097/QAI.0000000000002539 | The outcome was not recorded |
| 5 | Rawiwan Hansudewechakul, 2015 | Transition of Thai HIV-infected adolescents to adult HIV care. | Https://dx.doi.org/10.7448/IAS.18.1.20651 | The outcome was not recorded |
| 6 | Arantxa Berzosa Sánchez, 2021 | Mortality in Perinatally HIV-infected Adolescents After Transition to Adult Care in Spain. | Https://dx.doi.org/10.1097/INF.0000000000003009 | The outcome was not recorded |
| 7 | David Griffith, 2019 | Impact of a Youth-Focused Care Model on Retention and Virologic Suppression Among Young Adults With HIV Cared for in an Adult HIV Clinic. | Https://dx.doi.org/10.1097/QAI.0000000000001902 | Wrong population |
| 8 | Annette H. Sohn, 2020 | Peritransition Outcomes of Southeast Asian Adolescents and Young Adults With HIV Transferring From Pediatric to Adult Care. | Https://dx.doi.org/10.1016/j.jadohealth.2019.07.025 | The outcome was not recorded |
| 9 | Annouschka M. Weijsenfeld, 2016 | Virological and Social Outcomes of HIV-Infected Adolescents and Young Adults in The Netherlands Before and After Transition to Adult Care. | Https://dx.doi.org/10.1093/cid/ciw487 | The outcome was not recorded |
| 10 | Katherine Tassiopoulos, 2020 | Healthcare Transition Outcomes Among Young Adults With Perinatally Acquired Human Immunodeficiency Virus Infection in the United States. | Https://dx.doi.org/10.1093/cid/ciz747 | Wrong population |
| 11 | P. V. Safonova, 2023 | Adherence to treatment in young people with perinatal HIV infection in the first years after the transition from pediatric to adult medical service | Https://dx.doi.org/10.22328/2077-9828-2023-15-2-59-68 | Language |
| 12 | Brian C. Zanoni, 2020 | Transition from pediatric to adult care for adolescents living with HIV in South Africa: A natural experiment and survival analysis. | Https://dx.doi.org/10.1371/journal.pone.0240918 | Wrong study design |
| 13 | Patrick Ryscavage, 2016 | Linkage to and retention in care following healthcare transition from pediatric to adult HIV care. | Https://dx.doi.org/10.1080/09540121.2015.1131967 | Wrong population |
| 14 | JD Kowalska, 2019 | Both improvement and worsening of adherence to antiretroviral treatment can be expected while transitioning HIV-positive adolescents to adult health care. | Https://dx.doi.org/10.1080/23744235.2019.1582794 | The outcome was not recorded |
| 15 | Cosmina Gingaras, 2019 | Engagement in care among youth living with parenterally-acquired HIV infection in Romania. | Https://dx.doi.org/10.1080/09540121.2019.1612010 | The outcome was not recorded |
| 16 | Katarina Westling, 2016 | Transition of HIV-infected youths from paediatric to adult care, a Swedish single-center experience. | Https://dx.doi.org/10.3109/23744235.2016.1143964 | The outcome was not recorded |
| 17 | Qiang Xia, 2018 | Transition from pediatric to adult care among persons with perinatal HIV infection in New York City, 2006-2015. | Https://dx.doi.org/10.1097/QAD.0000000000001923 | Wrong population |
| 18 | Lana Lee, 2015 | Improved Retention in Care for Ugandan Youth Living With HIV Utilizing a Youth-Targeted Clinic at Entry to Adult Care: Outcomes and Implications for a Transition Model | Https://doi.org/10.1016/j.jadohealth.2014.10.101 | No full text is available |
| 19 | Ali Judd, 2017 | Growing up with perinatal HIV: changes in clinical outcomes before and after transfer to adult care in the UK. | Https://dx.doi.org/10.7448/IAS.20.4.21577 | The outcome was not recorded |

**Supplementary Table 3:** Quality assessment results of the included studies in the systematic review and meta-analysis based on the The Joanna Briggs Institute criteria (worldwide, 2024).

| **S. No** | **Included studies** | **Study design** | **Q-1** | **Q-2** | **Q-3** | **Q-4** | **Q-5** | **Q-6** | **Q-7** | **Q-8** | **Q-9** | **Q-10** | **Q-11** | **Total** | **Risk of bias** |
| --- | --- | --- | --- | --- | --- | --- | --- | --- | --- | --- | --- | --- | --- | --- | --- |
| **1** | Caroline FOSTER | Cohort | yes | yes | yes | yes | no | yes | yes | yes | yes | yes | yes | 10 | Low |
| **2** | Workabeba Abebe | Cohort | yes | yes | yes | yes | no | yes | yes | yes | yes | no | yes | 9 | Low |
| **3** | David Griffith | Cohort | yes | yes | yes | yes | yes | yes | yes | yes | yes | no | yes | 10 | Low |
| **4** | Sophia A. | Cohort | yes | yes | yes | yes | yes | yes | yes | yes | yes | no | yes | 10 | Low |
| **5** | Brian C. Zanoni | Cohort | yes | yes | yes | yes | no | yes | yes | yes | yes | no | yes | 9 | Low |
| **6** | Fatima Kakka | Cohort | yes | yes | yes | yes | no | yes | yes | yes | yes | no | yes | 9 | Low |
| **7** | Seema T. Meloni | Cohort | yes | yes | yes | yes | yes | yes | yes | yes | yes | no | yes | 10 | Low |
| **8** | Tanner Nassau | Cohort | yes | yes | yes | yes | yes | yes | yes | yes | yes | no | yes | 10 | Low |
| **9** | Paul Ouedraogo | Cohort | yes | yes | yes | yes | no | yes | yes | yes | yes | no | yes | 9 | Low |
| **10** | S. Hussen | Cohort | yes | yes | yes | yes | yes | yes | yes | yes | yes | no | yes | 10 | Low |
| **11** | Barbara Castelnuovo | Cohort | yes | yes | yes | yes | yes | yes | yes | yes | yes | no | yes | 10 | Low |
| **12** | Mary-Ann DavieS | Cohort | yes | yes | yes | yes | yes | yes | yes | yes | yes | no | yes | 10 | Low |
| **13** | Supattra Rungmaitree | Cohort | yes | yws | yes | yes | yes | yes | yes | yes | yes | no | yes | 10 | Low |
| **14** | Priscilla R Tsondai | Cohort | yes | yes | yes | yes | yes | yes | yes | yes | yes | no | yes | 10 | Low |
| **15** | Amanda E. Tanner | Cohort | yes | yes | yes | yes | yes | yes | yes | yes | yes | no | yes | 10 | Low |

Risk of bias

The Joanna Briggs Institute quality score ≥50%= Low risk of bias

The Joanna Briggs Institute quality score < 50%= High risk of bias

**Supplementary Table 4:** Meta-regression results of the systematic review and meta-analysis by study design (worldwide, 2024).

| _meta_es | Coefficient | Std. err. | z | P>z | [95% conf. | interval] |
| --- | --- | --- | --- | --- | --- | --- |
| Study design  **Retrospective Cohort** | .4296774 | .2034384 | 2.11 | 0.035 | .0309454 | .8284093 |
| _cons | 1.487835 | .3619258 | 4.11 | 0.000 | .7784738 | 2.197197 |

**Supplementary Table 5:** Eger’s test for publication bias in rate of retention after one year of transition: A systematic review and meta-analysis (worldwide, 2024).

| H0: beta1 = 0; no small-study effects | |
| --- | --- |
| beta1 | -0.95 |
| SE of beta1 | 2.036 |
| z | -0.47 |
| Prob > \|z\| | 0.6393 |

**Supplementary Table 6:** Eger’s test for publication bias in rate of retention after two years of transition: A systematic review and meta-analysis (worldwide, 2024).

| H0: beta1 = 0; no small-study effects | |
| --- | --- |
| beta1 | 5.90 |
| SE of beta1 | 4.476 |
| z | 1.32 |
| Prob > \|z\| | 0.1878 |
