# Supplementary material for: Retention in Care After Transition to Adult Care for Adolescents and Young Adults With HIV: A Systematic Review and Meta-Analysis
Source: Int J Public Health. 2025 Mar 24;70:1607733. doi: 10.3389/ijph.2025.1607733 (PMC11972947; doi:10.3389/ijph.2025.1607733)
Supplement: Supplementary file 2 [file DataSheet1.docx]

**Supplementary figures**

**Supplementary Figure 1:** Subgroup analysis of rate of retention one year after transition by geographic region: A systematic review and meta-analysis (worldwide, 2024)

**Supplementary Figure 2:** Subgroup analysis of rate of retention two years after transition by geographic region: A systematic review and meta-analysis (worldwide, 2024)

**Supplementary Figure 3**: Subgroup analysis by year of publication in the rate of retention after one year of transition: A systematic review and meta-analysis (worldwide, 2024)

**Supplementary Figure 4:** Subgroup analysis by year of publication in the rate of retention after two years of transition: A systematic review and meta-analysis (worldwide, 2024)

**Supplementary Figure 5:** Subgroup analysis by study design in rate of retention after one year of transition: A systematic review and meta-analysis (worldwide, 2024)

**Supplementary Figure 6:** Subgroup analysis by study design in the rate of retention after two years of transition: A systematic review and meta-analysis (worldwide, 2024)

**Supplementary Figure 7:** Subgroup analysis by type of population in rate of retention after one year of transition: A systematic review and meta-analysis (worldwide, 2024)

**Supplementary Figure 8:** Subgroup analysis by type of population in rate of retention after two years of transition: A systematic review and meta-analysis (worldwide, 2024)

**Supplementary Figure 9:** Subgroup analysis by median age of transition in rate of retention after one year of transition: A systematic review and meta-analysis (worldwide, 2024)

**Supplementary Figure 10:** Subgroup analysis by median age of transition in rate of retention after two year of transition: A systematic review and meta-analysis (worldwide, 2024)

 **Supplementary Figure 11:** Sensitivity analysis of included studies: A systematic review and meta-analysis (worldwide, 2024)

**Supplementary Figure 12:** Sensitivity analysis of included studies: A systematic review and meta-analysis (worldwide, 2024)
